# Effects of *Bacillus subtilis* supplementation on reproductive parameters during late gestation in multiparous sows

**DOI:** 10.14202/vetworld.2024.940-945

**Published:** 2024-05-04

**Authors:** Thepsavanh Khoudphaithoune, Do Thi Kim Lanh, Nguyen Van Thanh, Bui Van Dung, Bui Tran Anh Dao, Nguyen Hoai Nam

**Affiliations:** 1 Department of Animal Surgery and Theriogenology, Faculty of Veterinary Medicine, Vietnam National University of Agriculture, Vietnam; 2 Department of Veterinary Pathology, Faculty of Veterinary Medicine, Vietnam National University of Agriculture, Vietnam

**Keywords:** *Bacillus subtilis*, birth weight, pig, probiotic, stillbirth

## Abstract

**Background and Aim:**

Probiotics are used at different stages of gestation to promote reproductive performance in sows. This study investigated the effect of *Bacillus subtilis* QST 713 supplementation during late gestation in multiparous sows on different reproductive parameters.

**Materials and Methods:**

On day 85 of gestation, 115 multiparous healthy Landrace Yorkshire sows were randomly assigned to two groups with equal parity numbers. The control group (58 sows) was fed with basal diets, and the probiotic group (57 sows) was fed with basal diets +10^10^ colony-forming unit (CFU) *B. subtilis* QST 713 from day 85 to parturition. Back fat thickness on days 85 and 110, number of total born, number of born alive, stillbirth and mummy rates, individual birth weight, litter birth weight, within-litter variation of piglet birth weight, and postpartum vaginal discharge duration were recorded and compared between the two groups.

**Results:**

The number of total born, number born alive, back fat thickness of sows before farrowing, litter weight, within-litter variation of piglet birth weight, and postpartum vaginal discharge duration were similar in both groups (p > 0.05). Dietary supplementation with *B. subtilis* QST 713 decreased the stillbirth rate (3.96 vs. 6.39%, p = 0.046) and born dead rate (5.12 vs. 8.57%, p = 0.035) and increased the birth weight of piglets (1552.78 vs. 1506.15 g, p = 0.049).

**Conclusion:**

Daily supplementation with 10^10^ CFU of *B. subtilis* QST 713 during late gestation in multiparous sows could increase reproductive performance by increasing birth weight and decreasing stillbirth rate.

## Introduction

An increase in pig litter size leads to elevated stillbirth rates [1–3], reduced birth weight [4], and less uniformity of piglet birth weight [5–8]. Large litter sizes also result in deterioration in sows’ maternal ability [9], increased risks of prolonged postpartum vaginal discharge [10], and postpartum metritis [11]. These conditions increase animal welfare issues and lead to economic losses for the pig industry. In modern pig farming, the use of hyperprolific sows is irreversible. Therefore, approaches aiming at reducing stillbirth, increasing the birth weight and birth weight uniformity of piglets, and supporting the health of sows during the periparturient period may diminish the negative effects of large litter size selection.

Several probiotics have been supplemented during different stages of gestation to promote the reproductive performance and health of the sows [12–16], with controversial results. Some probiotics have been shown to increase the number of piglets born alive [17–20], litter birth weight [19, 21–23], and birth weight of piglets [21, 24]. On the other hand, some probiotics reduced the birth weight of piglets [13, 18, 25]. No effects of probiotics on such investigated reproductive parameters were detected in several other studies [12, 14–16].

*Bacillus subtilis* QST 713 is low toxic to animals and has been used to control necrotic enteritis in broilers [26] and to cope with dysbiosis in piglets [27]. This probiotic has been reported to increase the number of born alive, birth weight, and decrease the stillbirth rate of piglets when supplemented during late gestation in gilts [28].

In this study, we hypothesized that *B. subtilis* QST 713 would have beneficial effects on reproductive parameters in multiparous sows. Moreover, we evaluated the effect of *B. subtilis* QST 713 supplementation on back fat thickness before farrowing and duration of postpartum vaginal discharge in sows.

## Materials and Methods

### Ethical approval

The Committee on Animal Research and Ethics of the Faculty of Veterinary Medicine, Vietnam National University of Agriculture (CARE-2023/02) reviewed and approved the study protocol.

### Study period and location

This study was conducted from February to August 2023 on a farm in Quang Ninh province, Vietnam.

### Animals and housing

In total, 115 mixed parity Landrace Yorkshire crossbred sows (parity number 3.55 ± 0.87, 2–5) raised on one farm were used in the present study. During pregnancy, sows were kept in individual gestational crates and moved to farrowing rooms approximately 1 week before farrowing. Sows were allocated in individual farrowing crates measuring 2.2 × 0.6 m in farrowing rooms. During the first 21 days, sows were fed 1.8–2.4 kg of an industrialized gestating feed, which was increased to 2.0–2.8 kg during days 22–84 and 2.2–3.0 kg during days 85–107. Sows were fed 2.2–3.0 kg of industrialized lactating feed from day 108 to farrowing. The ingredients of gestating and lactating feeds consisted of soybean meal, animal protein, rice bran, rice, corn, cassava root, wheat bran, vitamins, amino acids, and minerals. Nutrient compositions of the feeds are presented in Table-1.

**Table 1:** Nutrient compositions of the basal diets.

Nutrient compositions	Gestation diet	Lactation diet
Crude protein (g/kg)	136	162
Crude fiber (g/kg)	62.2	60.2
Crude fat (g/kg)	78.2	66.7
Ash (g/kg)	59.6	60.6
Dry matter (g/kg)	894.2	895.3
Metabolizable energy (kcal/kg)	3585	3581
Total phosphorus (g/kg)	7.7	8
Calcium (g/kg)	9.6	9.1
Total amino acids (g/kg)	141.9	174
Selenium (mg/kg)	0.66	0.69
Copper (mg/kg)	47	53
Iron (mg/kg)	236	234
Zinc (mg/kg)	279	267
Manganese (mg/kg)	236	244

The industrialized gestation and lactation feeds comprised soybean meal, animal protein, rice bran, rice, corn, cassava root, wheat bran, vitamins, amino acids, and minerals. The gestational diet was fed to sows during the first 107 days of gestation. The lactational diet was fed to sows from day 108 of gestation to farrowing

Sows were vaccinated against classical swine fever, foot and mouth disease, and *Escherichia coli* at weeks 9, 12, and 14 of gestation, respectively. Vaccination against porcine reproductive and respiratory syndrome and Aujeszky’s disease was conducted every 4 months, and vaccination against porcine parvovirus was conducted on postpartum day 14. Sows were dewormed twice per year.

### Study design

Sows were randomly allocated into two groups on day 85 of pregnancy using a random number table. Randomization was conducted so that each group had a similar number of sows in each parity. According to this method, the treatment group had 6, 28, 15, and 8 sows in parities 5, 4, 3, and 2, respectively, and the control group had 6, 28, 15, and 9 sows in parities 5, 4, 3, and 2, respectively. In addition to the basal diet, sows in the treatment group were supplemented with 10^10^ colony-forming unit *B. subtilis* QST 713 (1 g, Baymix, GROBIG®, BS, Bayer de Mexico, S.A. de C.V., Mexico) per day. Supplementation was performed once per day in the morning. *B. subtilis* QST 713 was added to the feed of the sows so that it could be eaten. Sows in the control group consumed basal diets without *B. subtilis* QST 713 supplement.

### Data collection

Back fat thickness of the sows at both sites (site B) was measured on days 85 and 110 of pregnancy (Renco LEAN-MEATER, Renco, USA). The last rib of the sow was located by the middle finger, and site B, approximately 65 mm below the spine was identified. Cooking oil was applied to site B, and two measurements were conducted at each side. The back fat thickness of the sow was calculated as the average of four measurements. The parity number, day of insemination, and day of farrowing were recorded at farrowing. Litter size was the total number of piglets born, including those born alive and those born dead. Individual birth weight (g) of born alive piglets was measured before colostrum intake using a 5 g accurate portable digital hook weighing scale (Weihang, China). Litter birth weight was the sum of the birth weights of all piglets born alive. The mean birth weight of a given litter was calculated on the basis of the weight of individual live-born piglets in that litter. Within-litter variation in piglet birth weight (BWV) was expressed as either the standard deviation of birth weight or the coefficient of variation of birth weight. Standard deviation of piglets’ birth weight (g) was calculated from birth weight of individual piglets in a given litter. The coefficient of variation (%) of piglets’ birth weight was calculated by dividing the standard deviation by the mean birth weight of a given litter. After farrowing, postpartum vaginal discharge was observed twice per day in the morning and afternoon until no discharge was detected [10]. The postpartum vaginal discharge duration (PVDD) was calculated as the interval between farrowing and the first observation with no discharge.

### Statistical analysis

Independent Student’s t-test was used to compare back fat thickness, number of total piglets born per litter, number of piglets born alive per litter, standard deviation of birth weight, coefficient of variation of birth weight, and duration of postpartum vaginal discharge between the treatment and control groups. Chi-square test was used to compare the incidence of stillbirths, mummies, and born dead piglets in litter. Generalized linear mixed models were used to compare the stillbirth rate, mummy rate, and dead birth rate. The linear mixed effect model was used to compare individual birth weights. Student’s t-test and Chi-square tests were performed using the Statistical Package for the Social Sciences version 22.0 (IBM SPSS, Armonk, NY, USA). Generalized linear mixed models and linear mixed effect models were conducted in RStudio Desktop 1.3.1093 (RStudio Team: Integrated Development for R, Boston, MA, USA). A p < 0.05 was considered statistically significant.

## Results

A total of 1564 piglets were born from 115 investigated sows (13.6 piglets/Litter). Of the 1564 born piglets, 1458 were born alive (12.7 piglets/Litter), 106 were stillbirths, and 26 were mummies, resulting in rates of 5.1, 1.7, and 6.8% for stillbirth, mummy, and born dead, respectively. Individual birth weights were measured in 874 piglets born from 71 sows, including 33 sows in the control group and 38 sows in the treatment group. Back fat thickness of sows on days 85 and 110 of gestation was 14.6 ± 3.8 (range 7–26 mm) and 14.5 ± 3.8 mm (range 7–26 mm), respectively. The gestational length of the sows was 115.8 ± 1.8 days. The average piglet birth weight and standard deviation of birth weight were 1531.6 ± 348.2 and 250.6 ± 81.5 g, respectively. The litter birth weight was 18.76 ± 5.78 kg. The coefficient of variation of birth weight was 16.6 ± 5.9%. The duration of postpartum vaginal discharge in the investigated sows was 4.1 ± 1.8 days.

*B. subtilis* QST 713 supplementation from day 85 of gestation to farrowing did not influence the back fat thickness of the sows (p > 0.05). No difference was observed in litter size, number of born alive, mummy rate, percentage of litters with stillbirths, percentage of litters with mummies, percentage of litters with born dead piglets, duration of postpartum vaginal discharge, standard deviation of birth weight, and coefficient of variation of birth weight (p > 0.05) between the two groups. In contrast, *B. subtilis* QST 713 supplementation decreased the stillbirth rate (3.96 vs. 6.39%, p = 0.046) and born dead rate (5.12 vs. 8.57%, p = 0.035) and increased the birth weight of piglets (1552.78 vs. 1506.15, p = 0.049) (Table-2).

**Table 2:** Effect of *Bacillus subtilis* supplementation on different parameters in the investigated sows.

Investigated parameters	Control	Probiotic	p-value
Back fat thickness day 85 (mm)	14.69 ± 3.57 (n = 58)	14.45 ± 4.07 (n = 56)	0.741
Back fat thickness day 110 (mm)	14.60 ± 3.37 (n = 55)	14.37 ± 4.22 (n = 56)	0.753
Total born (piglets/litter)	13.48 ± 3.57 (n = 58)	13.72 ± 3.60 (n = 57)	0.724
Number of live-born piglets/litter (piglets)	12.31 ± 3.06 (n = 58)	13.11 ± 3.33 (n = 57)	0.185
Stillbirth rate (%)	6.39 (50/782)	3.96 (31/782)	0.046
Mummy rate (%)	2.17 (17/782)	1.15 (9/782)	0.225
Deadborn rate (%)	8.57 (67/782)	5.12 (40/782)	0.035
Percentage of litters with stillbirths (%)	37.93 (22/58)	35.09 (20/57)	0.752
Percentage of litters with mummies (%)	18.97 (11/58)	8.77 (5/57)	0.114
Percentage of litters with born dead (%)	44.83 (26/58)	43.86 (25/57)	0.917
Individual birth weight (g)	1506.15 ± 339.44 (n = 397)	1552.78 ± 354.29 (n = 477)	0.049
Litter birth weight (kg)	17.91 ± 5.93	19.49 ± 5.22	0.24
Standard deviation of birth weight (g)	255.33 ± 85.94 (n = 33)	246.55 ± 78.34 (n = 38)	0.587
Coefficient of variation of birth weight (%)	17.22 ± 6.34 (n = 33)	16.00 ± 5.43 (n = 38)	0.307
Postpartum vaginal discharge duration (day)	4.26 ± 1.54 (n = 58)	4.04 ± 1.94 (n = 57)	0.387

Values are mean ± standard deviation. Control: Basal diet treatment; Probiotic: basal diet + 10^10^ colony-forming units *Bacillus subtilis* QST713 treatment

## Discussion

This study showed that B. *subtilis* QST 713 supplementation during late gestation in multiparous sows could reduce the stillbirth rate and increase the birth weight of piglets. A positive effect of probiotics on birth weight was observed in the unaltered number of total born and number of born alive. Interestingly, although probiotics increased the birth weight of piglets, they did not affect the back fat thickness of the sows.

In the present study, the insignificant effect of probiotic on the number of total born and the number of born alive is corroborated by those found in several previous studies [12, 29-32]. In contrast, some authors found that the number of total born and the number of born alive in the probiotic groups were higher than those in the untreated groups [17–20, 28]. It is clear that probiotic supplementation during late gestation did not have any effects on the number of total born since this parameter was already fixed before probiotic use. However, it may alter the number of piglets born alive because this parameter depends not only on the number of total piglets born but also on the number of piglets born dead before or after the probiotic supplementation.

Although probiotic supplementation did not significantly increase the number of live births, it decreased the rates of stillbirth and dead birth. A previous study by Nam *et al*. [28] found that treatment with the same probiotic during late gestation in gilts decreased the stillbirth rate. In contrast, other authors have reported similar stillbirth rates between treatment and control groups [20, 22, 29, 33–35]. The decreased stillbirth rate in this study might be due to the increased birth weight of piglets in the probiotic groups (1552.78 vs. 1506.15 g) because the negative association between stillbirth rate and birth weight has been well established [2, 36–38]. All previous studies that did not observe a decrease in the stillbirth rate did not observe an increase in birth weight in the treatment group. However, the only study that found a decreased stillbirth rate also found an increased birth weight in the treatment group [28].

In this study, probiotics did not influence prepartum back fat thickness of sows. This finding is corroborated by the results of several previous studies [18, 23, 24, 31] in which probiotics were supplemented during a similar gestational period. Probiotics may improve the digestion and absorption of nutrients in sows. However, the potential beneficial effect of probiotics on back fat thickness during gestation cannot be expressed under feed restriction. Indeed, probiotic supplementation throughout gestation did not alter the back fat thickness of sows at farrowing [12, 21]. However, if supplementation was applied during lactation when the feed was provided *ad libitum* to appetite, it decreased the back fat loss of the sows [12, 21, 39].

Previous studies [12, 21, 24, 28] have found that probiotic treatment during gestation increases birth weight. On the other hand, other studies have reported that birth weight was not influenced by probiotic treatment [17, 29, 30, 32, 34, 40]. The birth weight of piglets in the treatment groups was lower than that in the control group [18, 35], and the decreased birth weight might be attributable to the increased litter size (14.94 vs. 13.13 piglets/litter) [18]. Differences in the results may be due to differences in probiotic strains, probiotic dose, nutrition content of feed, body condition score/back fat thickness, and gut microbiota of the sows in different studies.

This study did not observe any effects of probiotic treatment on BWV. Because measurement of individual piglet birth weight is time-consuming, information about the effect of maternal probiotic supplementation on this parameter is limited [21, 28]. In both of these studies, probiotic treatment failed to decrease BWV either during late gestation [28] or throughout two production cycles [21]. A negative association between BWV and mean birth weight has also been established [8]. In other words, BWV decreases when the birth weight increases. Although probiotics may increase the birth weight of piglets, this increment is too small to cause a change in BWV.

To the best of our knowledge, this is the first study to investigate the effect of probiotic supplementation during late gestation on PVDD in sows. Prolonged PVDD may result from severe trauma to the uterine tissue during farrowing and postpartum pathogenic infection [10, 41]. In women, oral supplementation of probiotics during late gestation prevents the decrease in *Bifidobacterium* and the increase in *Atopobium* as well as the decrease in antiflammatory cytokines, including interleukin 4 and interleukin 10 [42]. These results suggest that probiotics have a beneficial effect on the vaginal microbiota by increasing effective and decreasing pathogenic bacteria and reducing inflammation in the vagina. However, we failed to observe any beneficial effects of probiotic supplementation on the duration of postpartum vaginal discharge in sows. Future research should characterize the postpartum vaginal/uterine microbiota and inflammatory response in probiotic-supplemented sows to unravel the effect of probiotics on these parameters and postpartum reproductive diseases in sows.

## Conclusion

The findings of the present study indicated that supplementation with *B. subtilis* QST 713 from day 85 of gestation to farrowing decreased the stillbirth rate in multiparous sows, which may be attributable to increased birth weight.

## Authors’ Contributions

NHN, TK, DTKL, NVT, BVD, and BTAD: Conceived and designed the study. NHN and TK: performed the study, collected and analyzed data, interpreted the results, and drafted and revised the manuscript. NHN, TK, DTKL, NVT, BVD, and BTAD: Participated in scientific discussion. All the authors have read, reviewed, and approved the final manuscript.
